# Multi-Trait and Multi-Environment QTL Analyses for Resistance to Wheat Diseases

**DOI:** 10.1371/journal.pone.0038008

**Published:** 2012-06-05

**Authors:** Sukhwinder-Singh, Mateo V. Hernandez, Jose Crossa, Pawan K. Singh, Navtej S. Bains, Kuldeep Singh, Indu Sharma

**Affiliations:** 1 International Maize and Wheat Improvement Center (CIMMYT), Mexico Distrito Federal, Mexico; 2 Universidad Autonoma Chapingo, Chapingo, Mexico; 3 Department of Plant Breeding, Genetics and Biotechnology, Punjab Agricultural University, Ludhiana, India; Pennsylvania State University, United States of America

## Abstract

**Background:**

Stripe rust, leaf rust, tan spot, and Karnal bunt are economically significant diseases impacting wheat production. The objectives of this study were to identify quantitative trait loci for resistance to these diseases in a recombinant inbred line (RIL) from a cross HD29/WH542, and to evaluate the evidence for the presence loci on chromosome region conferring multiple disease resistance.

**Methodology/Principal Findings:**

The RIL population was evaluated for four diseases and genotyped with DNA markers. Multi-trait (MT) analysis revealed thirteen QTLs on nine chromosomes, significantly associated with resistance. Phenotypic variation explained by all significant QTLs for KB, TS, Yr, Lr diseases were 57%, 55%, 38% and 22%, respectively. Marginal trait analysis identified the most significant QTLs for resistance to KB on chromosomes 1BS, 2DS, 3BS, 4BL, 5BL, and 5DL. Chromosomes 3AS and 4BL showed significant association with TS resistance. Significant QTLs for Yr resistance were identified on chromosomes 2AS, 4BL and 5BL, while Lr was significant on 6DS. MT analysis revealed that all the QTLs except 3BL significantly reduce KB and was contributed from parent HD29 while all resistant QTLs for TS except on chromosomes 2DS.1, 2DS.2 and 3BL came from WH542. Five resistant QTLs for Yr and six for Lr were contributed from parents WH542 and HD29 respectively. Chromosome region on 4BL showed significant association to KB, TS, and Yr in the population. The multi environment analysis for KB identified three putative QTLs of which two new QTLs, mapped on chromosomes 3BS and 5DL explained 10 and 20% of the phenotypic variation, respectively.

**Conclusions/Significance:**

This study revealed that MT analysis is an effective tool for detection of multi-trait QTLs for disease resistance. This approach is a more effective and practical than individual QTL mapping analyses. MT analysis identified RILs that combine resistance to multiple diseases from parents WH542 and/or HD29.

## Introduction

Among biotic stresses, stripe rust (Yr) (*Puccinia striiformis* f. sp. *tritici*), leaf rust (Lr) (*P. triticina*), tan spot (TS) (*Pyrenophora tritici-repentis*), and Karnal bunt (KB) (*Tilletia indica*) are important diseases that adversely affect yield and quality of bread wheat (*Triticum aestivum* L.) throughout the world. Regional differences in severity and incidence are pronounced for these diseases. Among these, KB is very difficult to control once it is introduced into an area and its potential impact on the grain industry remains high because of quarantine against the disease. Direct and indirect losses caused by KB in northwestern Mexico and in the northern Texas State in the USA were projected at US $7 million and $25 million per year respectively [1]–[2].

The identification and introgression of broad genetic base resistance in commercially grown wheat cultivars is the most cost effective and environmentally safe means to manage wheat diseases. Most single gene resistances against pathogens of wheat as well as many other crops have proven to be non-durable [3]–[4]. Inheritance of resistance for Yr and Lr, diseases of wheat is both qualitative and quantitative however for TS and KB are mainly quantitative [5]–[9]. Analyses of QTL were reported in number of studies for resistance to wheat pathogens [10]–[12]. Multiple disease resistance (MDR) loci to many pathogens in wheat have been identified [13]–[14]. Important example is the *Lr34/Yr18/Pm38/Bdv1* locus which confers resistance to leaf rust, yellow rust, powdery mildew, and barley yellow dwarf virus. This locus represents a single gene [15] and the gene has been cloned. QTLs for Septoria tritici blotch (STB) Stagonospora nodorum blotch (SNB), and Fusarium head blight (FHB), diseases of wheat has been reported [16]. Defense related genes in wheat have been reported not randomly distributed throughout the wheat genome, but in clusters and/or in distal gene-rich regions of the chromosomes [17]. MDR have also been reported in Arabidopsis, maize and rice [18]–[20].

Quantitative resistance is controlled by minor genes with small additive effect, and is more durable. For wheat rusts, adult plant resistance (APR) tends to slow the development of the disease rather than providing immune reactions by preventing the disease development. Genetic control of APR reaction has been reported to be controlled by minor genes [21]. This is thought to result from the host's ability to lengthen the time required for the pathogen to colonize and to reduce sporulation capacity of the pathogen.

Identification and genetic characterization of new sources of resistance and their transfer to adapted genetic backgrounds is of great importance for wheat improvement. The development of molecular markers closely linked to resistance QTLs offers alternative methods for selection of resistant germplasm, facilitates effective pyramiding of resistance QTLs and offers the possibility of selecting resistant genotypes in the absence of the pathogens [9], [15], [22]–[26]. The availability of DNA markers provides an additional means to determine gene uniqueness. Apart from their indirect use in pyramiding resistance genes throughout marker assisted selection, markers also help to verify findings of conventional analyses, which become complicated when large numbers of genes are already known. Such a situation is encountered in the case of rusts of wheat where more than 160 resistance genes are named [27].

The objective of this research was to examine multi-trait or multi-environment QTLs analysis, to identify chromosome regions with MDR in wheat, and identify marker-phenotype associations for diverse traits with a data set of RIL population in wheat.

## Materials and Methods

### Plant materials

The RIL population was developed following single-seed descent of individual F_2_ plants to F_6_ followed by further generations of advance using bulked samples. The population comprised of 109 RILs derived from the cross WH542/HD29. Both the parents are from Indian spring wheat pool. HD29 is resistant to KB but susceptible to rusts and TS whereas WH542 is susceptible to KB but resistant to rusts and TS. WH542 is sister line of a widely adapted CIMMYT breeding line Kauz (Jupateco/Bluejay//Ures).

### Disease screening

RILs were grown in 1 m long pair row plots with row–to-row and plant-to-plant distance of 23 cm ad 10 cm, respectively. Likewise, parents were planted in four-row plots. The RILs and the parents were grown in a completely randomized design with three replications over five years. The RIL population was screened for KB resistance during five years (2000-01, 2001-02, 2002-03, 2003-04 and 2004-05) as described by Singh et al. [9]. Correlation coefficients (*r*) among years were estimated on the adjusted means of the RILs.

Field evaluations for Lr and Yr reaction were conducted at Punjab Agriculture University, Ludhiana, India, during crop season 2004-05. Parental genotypes were included as controls. The HD29/WH542 RIL population was sown as 60 cm rows in the field. A boarder row of susceptible infector wheat surrounded the experimental material for uniform disease development. Urediniospores of different rust pathotypes suspended in light mineral oil were misted over spreader rows and the experimental rows using an ultra low volume applicator. Rust susceptible spreader rows served as inoculum source for epidemic development in addition to infection from direct inoculation of the experimental rows. Variation in adult plant rust response was recorded independently in Yr and Lr trials. Disease severity on parents and the RILs was scored according to the modified Cobb Scale where percentage of rusted tissue was visually estimated according to Peterson et al. [28]. Rusts response assessments were performed when the susceptible parent reached 100% rust severity. RILs were also evaluated against TS as described previously Singh et al. [29]. WH542 is resistant and HD29 moderately susceptible to race 1 of *P. tritici-repentis*. The reactions of RILs, and the two parents to tan spot were determined in greenhouse experiments as described previously [29].

### Marker genotyping

Leaf tissue was harvested from each RIL and the parents. Tissue was ground in liquid nitrogen and genomic DNA was extracted using the CTAB-DNA method as described Singh et al. [9]. Polymorphism between parents was assessed with PCR-based DNA markers including SSRs and EST-STS markers (http://wheat.pw.usda.gov/cgi-bin/westsql/map image.cgi). PCR conditions, running and scoring gels as mentioned previously [29]. Genetic linkage maps were constructed with MAPMAKER version 2.0 for Macintosh [30]. Markers within groups were ordered at LOD 3.0.

### QTL analyses

Mixture and Mixed models were used, for both data sets; multi-environment QTL analysis (data set with KB evaluated in five years, KB2001M, KB2002M, KB2003M, KB2004M, and KB2005M) and multi-trait QTL analysis (with traits KB, TS, Yr, and Lr). For the multi environment and multi-trait QTL analyses the mixed models framework in the procedure QMQTLSCAN implemented in the Genstat release 13 [31]–[32] was used.

### Mixed model for single trait multi environment or single environment multi trait QTL analysis

The basic phenotypic model for a single trait multi environment (or single environment multi trait) can be expressed as:
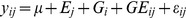
(1)where *y_ij_* is the trait value of genotype *i* in environment (or trait) *j*, *E_j_* is the environmental (or trait) main effect, *G_i_* is the genotypic main effect, *GE_ij_* is the genotype by environment interaction, and *ε_ij_* are the random errors, assumed to be normally and independently distributed with mean zero and homoscedastic variance *σ ^2^*.

When the additive effects of the molecular markers information is considered the model becomes:
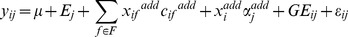
(2)if both additive and dominance effects are specified the model is
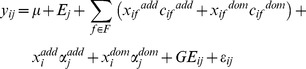
(3)where F is a set of cofactors (if cofactors are included in the model), 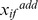 and 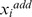 are the additive genetic predictors of genotype *i* at the cofactors position and at the tested position, respectively. The associated effects are denoted by 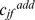 and 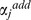 for cofactors and tested position respectively. In model 3, 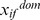 and 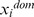 are dominance genetic predictors of genotype *i* at the cofactor position and at the tested position, respectively, with associated effects 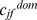 and 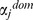.

Genetic predictors are genotypic covariables that reflect the genotypic composition of a genotype at a specific chromosome location. The residual unexplained genetic and environmental effects are modeled by the *GE_ij_* term, which is assumed to follow a multi-Normal distribution with mean vector 0 and a variance covariance matrix Σ. The matrix Σ can either be modeled explicitly (with an unstructured model) or by some parsimonious model.

Both, the multi-environment and multi-trait QTL analyses can be seen as particular cases of the more general mixed model for multi-trait multi-environment (MTME) data [33].

### Mixed model for multi environment (ME) or (multi trait, MT) data using matrix notation

Following Malosetti et al. [33], consider a ME (MT) data set consisting of I genotypes, evaluated in J environments (traits). Define an N × 1 vector **y** with N = IJ containing all the observations sorted by environment (trait) within genotype. In the linear model, random variables will be underlined. Given that the interest is in the genetic variation within the population rather than the genotypes themselves, we assume genotypes to be random, whereas the environments (traits) as well as other design factors are taken as fixed effects in order to minimize the environment to environment mean differences. A general formulation of a mixed model for the ME (MT) data is:
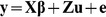
(4)


The response trait is represented in vector **y** and it is modeled by a set of fixed effects collected in vector **β** and random effects collected in vectors **u**, and **e**. **X** and **Z** are design matrices assigning the fixed and random effects, respectively to the observations. Vector **β** contains the trait means within environments (traits) across genotypes. Vector **u** denotes the random genotypic effects per environment (trait). Random genetic effects are assumed to be normally distributed, **u∼**N (0, **G**); with **G** being the genetic (co)variance matrix. Finally, **e** is a vector of non-genetic residuals associated with each observation and normally distributed, **e**∼N (0, **R**) with **R** being the residual variance. The phenotypic (co)variance is given by
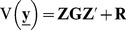
(5)


For a ME (MT) model given by (4) the **G** matrix has in its diagonal the genetic variance of each trait-environment combination (i.e. 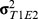 for trait *T1* in environment *E2*) and in its off-diagonal the genetic covariance between each pair of trait-environment combinations (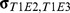 for trait *T*1 in environment *E*2 with *T*1 in environment *E*3 or 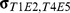 for trait *T*1 in environment *E*2 with *T*4 in environment *E*5) [33].

From breeders' point of view, the covariance matrix is of special interest as it reflects the magnitude and pattern of relationships between genetic effects. Random genetic effects across a set of environments will not be independent if there are genes/QTLs with effects across those environments; similarly genetic effects for different traits are not independent if genes/QTLs for different traits are linked or pleiotropic. The effect of genes/QTLs across environments (traits) will often not be equal in size, and sometimes not even in sign, leading to heterogeneous genetic variances. The model for covariance matrix should reflect these relationships and the heterogeneities in genetic variation.

A QTL model arises from Eq. 4 by including the effect of a putative QTL as follows:
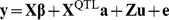
(6)


The extra term in the model is composed of a design matrix **X** ^QTL^, which is derived from molecular marker information (a further description of this key matrix will follow), and a vector of fixed QTL effects (**α**). In an ME (MT) model, vector **α** contains the additive genetic QTL effects for all the environments (traits). The random genetic effects are collected in vector **u** and result from the effects of QTLs outside the tested region, that is, the genetic background. Genetic background effects are assumed normally distributed: **u**∼N (0, **G**). Note that **G** represents the part of the genetic (co)variance that is not explained by the QTL.

The extension from a single QTL model to a multi QTL model is straightforward and is given by
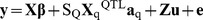
(7)


The QTL section includes the additive effects of all detected QTLs in the genome. The values of the Wald statistics or the associated tail probabilities, P, expressed as -log_10_ (P), serve to produce plots analogous to the usual LOD score profiles in QTL mapping. By plotting the -log_10_(P) along the chromosomes, we identified putative QTLs at those positions for which peaks in the profile exceeded a threshold value. We used a Bonferroni-based multiple test control threshold, using the estimation of the effective number of tests along the genome proposed by Li and Ji [34]; as shown by the authors in simulation data, this test is efficient and accuracy and it provides an alternative to the permutation test We control the genome-wide alpha level at 0.05, which corresponded to a point-wise alpha level of 0.05 divided by the effective number of tests along the genome. For our data the threshold found and used was 3.38, which corresponds to a point wide alpha equal to 0.00042

### QTL mapping: scanning and testing procedure

The mixed model strategy used consisted of three steps. In the first step, a phenotypic mixed model was fitted to genotype by environment data, where the aim was to identify a variance covariance model. At this stage, no marker information was included in the model; this includes model 1 or model 4. Some variance covariance structures that can be used are:

- Compound symmetry
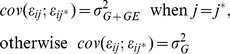

- Heterogeneous compound symmetry
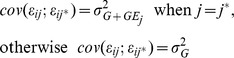

- First-order analytic factor + heterogeneity
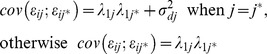

- Second-order analytic factor + heterogeneity
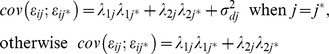

- Unstructured
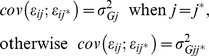

The simplest model, (i), is the compound symmetry model that requires the (residual) genetic variance, 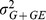, to be the same in all environments, and the (residual) genetic covariance be constant across all pair of environments. Somewhat less simple is the heterogeneous compound symmetry model (ii) that allows the genetic variance to differ between environments, while retaining the property of constant covariance across all pairs of environments. The most flexible, model (v), the unstructured model, gives each environment its own genetic variance and each pair of environments its own genetic covariance. Almost as flexible as model (v), but requiring less parameters are models (iii) and (iv). The factor analytic model with one and two multiplicative terms, with *λ*_1*j*_ and *λ*_2*j*_ environment specific multiplicative parameters and 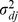 a residual heterogeneity [39]. Because in this study the number of environments (traits) and genotypes are relatively small, we used directly the most flexible variance covariance unstructured model.

In the second step we performed a repeated genome scan for the detection of environment-specific QTL effects. The first genome scan for QTL corresponded to simple interval mapping [35], in which a putative QTL is moved along the genome and at each position; a test for environment-specific QTL is performed. The mixed model that we used to test for environment specific QTL contained marker related information (genetic predictors) in the fixed part of the model, combined with the variance-covariance structure between environments identified in the previous phenotypic analysis (model 2 or model 6).

The third step consists in a second scan, where the genetic predictors of identified QTL of the first scan were used as cofactors. This second scan was performed by multi environment composite interval mapping. Jiang and Zeng [36] proposed a comparable procedure in a mixture model context. Also in this third step of our procedure, for the identified QTL positions in the last genome scan, we fitted a multi QTL model using a backward selection procedure in order to obtain the final significant QTLs and the estimation of their effects in each of the environments (model 2 or model 7).

### Mixture model for multi environment (ME) and multi trait (MT) data

The mixture model framework is similar to the mixed model framework established in Eqs. 4 to 7, the difference is that the term **Zu** is not included, whereas all the other terms are considered as fixed effects, except the residuals. That is, the model without including marker information is:
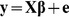
(8)where the mean of each of the terms is the same as in eq. 4 . The model including a putative QTL, becomes:
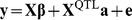
(9)and finally the model including multiple QTLs will be:
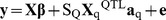
(10)


For this approach we have used the software QTL Cartographer [37], specifically the JZmapqtl option which implements simple interval and composite interval mapping for multiple environments (traits). JZmapqtl can jointly analyze more than one environment (trait). It is best used when one suspects that two environments (traits) are correlated. JZmapqtl creates a number of different output files depending on the number of environments (traits) in the joint analysis. There will be one file per environment (trait) that has estimates for the parameters for that environment (trait) and there will be one other file that contains the results of the joint likelihood ratio. One special case of G × E analysis has been incorporated into JZmapqtl, namely the situation where a set of genotypes is raised in more than one environment. The value of the trait in each environment is treated as a separate trait for the common genotype.

We used the stepwise regression analysis option with 0.05 alpha level in both, input and output, for selecting the putative QTLs to be used as cofactors later in the joint composite interval mapping (JZmapqtl option). In this last option we used a windows size of 30 cM for blocking the markers effects, others than the position being tested on the same chromosome.

In Cartographer it is possible to perform the permutation test [9] with the aim of estimating experiment specific threshold values for each individual environment (trait) and for the joint analysis. The threshold values were determined using 1000 permutations.

### Other software used for QTL analysis

Also we have performed the QTL analyses employing a program package written in FORTRAN language, which has been routinely used in CIMMYT from several years. These programs are very similar to QTL Cartographer software. However, one advantage of the CIMMYT programs compared to the other software is that it is easier to control the genetic background or cofactors. Similar to JZmapqtl in Cartographer, first we run a simple interval mapping looking for the putative QTLs to be used as cofactors in composite interval mapping. Then we run restricted composite interval mapping with a window size greater than the largest chromosome, for detecting possible ghost or new QTLs, determining the final cofactors set. Finally run a second composite interval mapping with the final cofactors found previously and a window size of 30 cM. The threshold value was established using a fixed criterion, based on a chi-square distribution with degrees of freedom depending on the number of environments (traits) analyzed simultaneously.

Similar to QTL Cartographer, in the CIMMYT programs the output generates the marginal as well as the joint likelihood ratio profiles, additive estimates, and the QTL by Environment Interaction, so it is possible to obtain the marginal and joint graphs. Also using this programs it is possible to calculate the R^2^ values or the phenotypic proportion of variance explained by each QTL found in each individual environment (trait).

We compared the results obtained using the different approaches and these provide similar outcome in terms of QTL detection. We present the results obtained in the last strategy, within a mixture model framework, because they allow to draw the marginal and the joint likelihood profile together, as well as to obtain the estimates of additive and proportion of phenotypic variance explained by each QTL in each environment (trait).

The threshold value was determined using 1000 permutations [38]. In the tables we are reporting the values corresponding to the peak for the joint profile, as well as the peaks for the marginal traits profiles. Therefore, often in the figures one significant peak for joint profile could be no significant for some marginal trait profile, so in the table the LOD appear with values lower than the threshold; usually LOD value of the peak for the marginal traits are slightly shifted from the peak of the joint analysis.

## Results and Discussion

**Figure 1:**
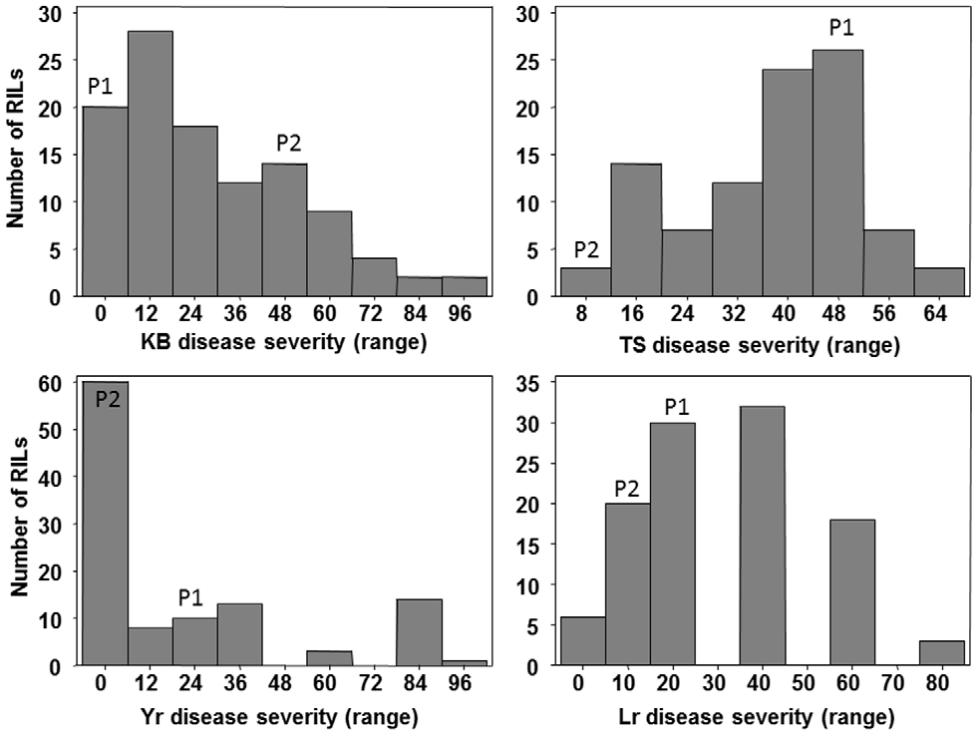
Disease frequency distribution of KB, TS, Yr, and Lr in the RIL population HD29/WH542. The mean value of the parents HD29 (P1) and WH542 (P2) is also shown on the figure.

The objectives of this study were to map QTLs for four different diseases of wheat and to identify chromosomal regions harboring resistance to the multiple diseases in a mapping population using multi-trait-analysis. The RIL population HD29/WH542 was analyzed for quantitative resistance for four different pathogens: *P. striiformis* f.sp. *tritici* (Yr), *P. triticina* (Lr), *P. tritici-repentis* (TS), and *T. indica* (KB). Evaluation of disease resistance in the RIL population exhibited continuous distribution for KB, TS, and Lr diseases. Fig. 1 show the frequency distributions for KB, TS, Yr, and Lr diseases and resistance was hypothesized to be quantitatively inherited for all except Yr, as described in earlier reports [8]–[9], [29]. The parental lines, HD29 and WH542, differed for response to all disease traits evaluated, except for Yr. Despite intermediate adult plant reaction to Yr for both the parents, the RIL population segregated for this disease (Fig. 1) indicating genetic independence of Yr resistance genes carried by these parents. Results revealed significant variation (P<0.001) among genotypes and genotype-by-environment interactions for all traits (data not presented). KB was found to be significantly negatively correlated with TS (Table 1), this is the reason why in the putative QTLs found generally both profiles were similar, the negative correlation can be observed in the opposite sign for the additive effect for most of the QTLs. KB resistance did not show any significant correlation with Yr and Lr. Similarly Yr and Lr were significant, but negatively correlated, and again this correlation can be observed in the similar profiles for the putative QTLs 3, 5, and 9 and except the first QTL in all the other chromosomes they shown opposite signs for the additive effects. TS and Yr were also significantly and negatively correlated, which was reflected in the additive effects opposite sign for eleven out the thirteen putative QTLs found.

**Table 1:** Phenotypic correlations among mean score for Karnal bunt (KB), tan spot (TS), yellow rust (Yr), and leaf rust (Lr) diseases.

Trait	KB	TS	Yr
**TS**	−0.3424***		
**Yr**	0.0550 NS	−0.3275***	
**Lr**	0.0823 NS	0.1448 NS	−0.3294***

*** : Significant at P≤0.001 alpha level.

NS: Non significant at P≤0.05 alpha level.

Table 2 shows the genotypic and phenotypic correlations for KB across the five years. It is interesting to note that the genetic and phenotypic correlations were very similar in value, and all the years were positively correlated, indicating the consistency of their values. Their positive correlation was reflected in the additive effect sign for the three putative QTLs detected, where except two out 18 additive effects all were negative (HD29), both for the marginal and joint profile. Particularly in the couples of years 2001 with 2002 and 2003 with 2004 were highly correlated.

**Table 2:** Genetic (lower diagonal) and phenotypic (upper diagonal) correlations for Karnal bunt (KB) across the different years.

Trait†	KB01	KB02	KB03M	KB04M	KB05M
**KB01**	1	0.8665*	0.4961*	0.3771*	0.3415*
**KB02**	0.8721*	1	0.5832*	0.4396*	0.3619*
**KB03M**	0.4798*	0.5521*	1	0.6783*	0.5259*
**KB04M**	0.4071*	0.4680*	0.7688*	1	0.4318*
**KB05M**	0.3460*	0.3664*	0.5162*	0.4672*	1

† : KB01, KB02, KB03, KB04, and KB05 indicate Karnal bunt disease score for year 2001, 2002, 2003, 2004, 2005, respectively.

* : Significant at P≤0.001.

### Multi-trait QTL

**Figure 2:**
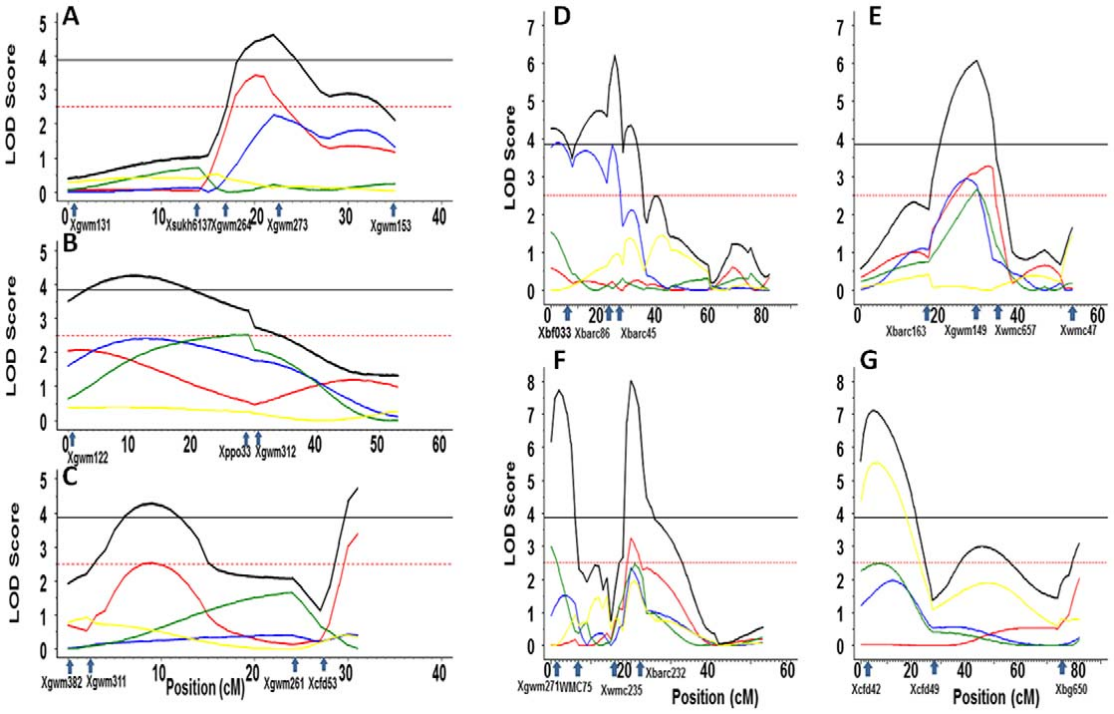
Multi-trait analysis and locations of QTL for resistance to KB, TS, Yr and Lr. (**A**) chromosome 1BS with marker *Xsukh6137* (at 14 cM), marker *Xgwm264* (at 17 cM) and marker *Xgwm273* (at 22 cM) where the QTL are located; (**B**) chromosome 2A with marker *Xgwm122* (at 0 cM), marker *Xppo33* (at 29 cM where the QTL is located), and marker *Xgwm312* (at 30 cM); (**C**) chromosome 2DS with marker *Xgwm311* (at 2 cM) and marker *Xgwm261* (at 24 cM); (**D**) chromosome 3AS with marker *Xbarc86* (at 21 cM), marker *Xbarc45* (at 24 cM where the QTL is located), *Xbem29* (at 27 cM), and *Xbem7* (at 35 cM); (**E**) chromosome 4BL with marker *Xbarc163* (at 18 cM), marker *Xgwm149* (at 28 cM where the QTL is located), marker *Xwmc657* at 34 cM; (**F**) chromosome 5BL with marker *Xgwm271* (at 2 cM where the QTL is located), marker *Xwmc75* (at 6 cM), marker *Xwmc235* (at 20 cM) and marker *Xbarc232* (at 22 cM) and (**G**) chromosome 6DS with marker *Xcfd42* (at 0 cM where the QTL is located), and marker *Xcfd49* (at 27 cM). LOD scores are plotted against marker location. QTLs profiles legend with color red, blue, green, yellow, and black indicate KB, TS, Yr, Lr, and joint effect respectively. Horizontal lines with black solid color mean threshold for Joint profile, and red dashed is threshold for marginal profiles.

In majority of mapping studies, data were recorded for several traits, and analyzed independently for each trait [5]. Thus, it is not possible to distinguish between pleiotropy and linkage of genes as underlying causes of genetic relationship between traits. As a result, only partial information about the genetic architecture of the traits under consideration is discovered. Multi-environment and multi-trait QTL mapping approaches have been proposed previously [39]–[40]. Malosetti et al. [41] proposed an approach by integrating molecular markers into the linear mixed model methodology which we applied in this study as well as the other two approaches described above. For the mixture model approach we detected thirteen putative QTLs on nine different chromosome regions (1BS, 2AS, 3DS, 3AS, 3BS, 4BL, 5BL, 5DL, and 6DS) (Table 3) for MDR in wheat. These chromosomes have also been reported to be associated with disease resistance in numerous genetic studies in wheat [5], [6], [9], [11], [24]–[26], [29], [42]. Present investigation support those results and it may be possible that these regions in wheat genome provide general defense against pathogens. In all the QTLs, at least one marginal trait was found to be significant (threshold = 2.5), except in chromosome 5DL-1, in which the maximum marginal peak was lower than 2.5 (2.26) for KB trait. However, in this chromosome joint analysis was significant. In other chromosomes, the peak for marginal traits was found similar with the joint profile peak. Marginal trait KB was significant on chromosomes 1BS, 2DS.1, 2DS.2 3BS, 3BL, 4BL, 5BL.2 and 5DL.2 (Table 3). Chromosome 1BS was found to have a LOD value of 3.41 for KB trait at the 20 cM position (Fig. 2 A), whereas for the joint analysis it was observed at 22 cM position. In almost all the QTLs reported in Table 3, the behavior for marginal traits and joint profile was very consistent for all except for yellow rust (*QYr.cimmyt-2AS*). KB was the trait which showed the maximum LOD values 5.60 at 14 cM position and probably was the trait that influences more the joint behavior on chromosomes 1BS, 2DS, 3BS, 4BL, 5BL and 5DL. The most significant joint profile peak was observed at 20 cM (LOD 7.99), on chromosome 5BL-2 and it may be more influenced by KB trait.

**Table 3:** First part: Significant QTLs, chromosome, nearest marker, LOD score values (outside brackets, in bold) and their positions (inside brackets, in italics) for multi trait analyses including traits Karnal bunt (KB), tan spot (TS), yellow rust (Yr) and leaf rust (Lr).

	LOD Score						
QTL	Chr	Marker	KB†	TS	Yr	Lr	JOINT‡
**1**	1BS	*Xgwm273*	**3.41**(*20*)	**2.25**(*22*)	**0.72**(*14*)	**0.53**(19*)*	**4.61**(*22*)
**2**	2AS	*Xgwm122*	**2.07**(*2*)	**2.41**(*12*)	**2.53**(*29*)	**0.39**(*8*)	**4.26**(*11*)
**3**	2DS.1	*Xgwm311*	**2.53**(*9)*	**0.40**(*24)*	**1.66**(*24*)	**0.93**(*2*)	**4.27**(*9*)
**4**	2DS.2	*Xcfd53*	**3.40**(*31)*	**0.42**(*30)*	**0.51**(*28*)	**0.41**(*30*)	**4.71**(*31*)
**5**	3AS	*Xbarc45*	**0.58**(*0*)	**3.84**(*23*)	**1.53**(*0*)	**1.44**(*41*)	**6.20**(*24*)
**6**	3BS	*Xgwm285*	**5.60**(*14*)	**1.85**(*9*)	**0.27**(*5*)	**1.46**(*3*)	**6.38**(*13*)
**7**	3BL	*Xgwm340*	**2.62**(*94*)	**1.36**(*94*)	**1.97**(*94*)	**0.04**(*94*)	**4.79**(*94*)
**8**	4BL	*Xgwm149*	**3.29**(*32*)	**2.93**(*26*)	**2.66**(*29*)	**0.10**(*23*)	**6.07**(*29*)
**9**	5BL.1	*Xgwm271*	**0.17**(*6*)	**1.50**(*4*)	**3.00**(*0*)	**1.45**(*12*)	**7.72**(*2*)
**10**	5BL.2	*Xwmc235*	**3.25**(*20*)	**2.96**(*20*)	**2.47**(*21*)	**1.94**(*21*)	**7.99**(*20*)
**11**	5DL.1	*Xest-002*	**2.26**(*6*)	**1.66**(*7*)	**0.38**(*6*)	**0.42**(*6*)	**3.88**(*6*)
**12**	5DL.2	*Xgwm90*	**3.92**(*25*)	**0.55**(*18*)	**0.01**(*24*)	**0.96**(*24*)	**5.78**(*24*)
**13**	6DS	*Xcfd42*	**0.03**(*5*)	**1.96**(*12*)	**2.48**(*6*)	**5.53**(*6*)	**7.10**(*5*)

	Additive effects							R^2^	
QTL	KB	TS	Yr	Lr	JOINT	KB	TS	Yr	Lr
**1**	−5.91	4.17	5.18	−2.77	−0.05	11.35	13.40	1.53	0.48
**2**	−4.70	4.88	−11.04	2.74	−0.01	17.57	24.20	4.02	1.18
**3**	−10.76	−1.81	8.92	4.93	−0.73	13.16	1.57	3.44	1.29
**4**	−7.46	−2.62	5.36	−4.31	−3.78	16.17	0.91	1.53	0.22
**5**	−1.93	5.75	−7.51	−9.16	1.85	3.01	22.74	7.74	0.27
**6**	−7.71	3.78	−4.75	6.01	−0.25	18.75	12.14	0.07	0.48
**7**	5.36	−2.93	11.20	−0.52	0.98	0.18	0.08	12.52	2.28
**8**	−5.62	5.25	−10.03	2.78	−0.67	14.47	6.60	6.85	0.05
**9**	−1.54	4.68	13.20	6.99	3.51	3.70	9.78	8.05	0.06
**10**	−4.88	4.04	−11.47	6.41	−0.11	12.10	8.49	2.79	4.81
**11**	−4.01	3.25	−3.82	2.52	0.12	17.92	10.88	0.33	0.84
**12**	−6.57	2.35	−4.00	5.28	−0.32	17.59	4.73	0.08	1.50
**13**	−1.03	4.67	−12.13	11.66	1.16	3.82	10.37	2.25	8.63
	R^2^ Total					56.57	55.22	38.21	22.32

Second part: Additive effect of the QTL and R^2^ for each trait. For the individual trait profiles, the LOD values are those found at the maximum individual peak.

† : Average threshold for individual trait analysis was 2.5.

‡ : Threshold for Joint analysis was 3.87.

For chromosome 3AS, the trait influencing joint profile the most was TS and both marginal and joint profile are located at very similar positions (Table 3 and Fig. 2 D). In chromosome 3AS (*QTs.cimmyt-3AS*) the joint profile peak for TS was detected at 24 cM position with a LOD of 6.20 while for the marginal traits the maximum peak LOD 3.84 was observed at 23 cM position (Fig. 2 D). Trait TS was also significant at the chromosome 4BL, but shifted around the 26 cM (LOD 2.93) from the peak of the joint profile at 29 cM (LOD 6.07).

The disease Yr, was found significant on chromosomes 2AS, 4BL and 5BL-1 (Table 3). In chromosome 2AS (*QYr.cimmyt-2AS*) the peak for the joint profile was at 11 cM while the maximum peak for Yr was detected at the 29 cM with a LOD of 2.53 (Table 3, Fig. 2 B). Lr was found significant (LOD 5.53) only on chromosome 6DS at 6 cM position, and this trait influenced joint profile the most. While the other three traits had a LOD value lower than 2.5. However, note that all the four individual traits showed their maximum tendency in around the same peak of the joint profile, i.e. around 5 cM (Fig. 2 G). MT method allows studying the occurrence of QTL by environment interaction; it facilitates examining the causes of genetic correlations between traits which results from either linked QTLs or pleiotropic QTLs. Further, it determines the changes in genetic correlations between traits across environments, which are caused by linked or pleiotropic QTLs showing QTL by environment interaction.

Interestingly, the resistance locus *QKb.ksu-4BL* for KB identified in HD29 in a previous study [9] and in this study, resides in the same region as resistance for tan spot and yellow rust diseases. There are evidences for the existence of MDR QTLs, however, they were detected independently from the crop and the pathosystem. The question is whether this reflects just a random co-localization of resistance genes in gene rich genomic regions or the action of the same gene on different pathogens. Poland et al. [14] discussed several hypotheses for explaining the potential mechanisms underlying MDR QTLs. The most probable hypothesis for MDR in this study is their involvement in basal defense reaction or defense signal transduction. Because the pathogens analyzed are not genetically related and infect different plant organs (leaf vs. head) in different adult-plant stages (flag leaf extension vs. flowering), the hosts' resistance reaction might be triggered by a widely conserved pathogen elicitors. Molecular studies in *Arabidopsis* support the hypothesis that pattern-recognition receptors can condition quantitative differences in resistance to several pathogens [43]–[44].

### Individual QTL analyses

In order to compare and verify the consistency of the marginal QTL results obtained on the multi-trait analysis, we performed analysis for individual traits; KB, TS, Yr, and Lr, one at a time. The individual and marginal profiles were very similar not only in the chromosomes in which the significant QTLs were found but also in all the other chromosomes (data not shown). In some QTLs, the LOD score in the marginal profile was greater than in the individual profile, while in others cases the behavior was the opposite. For example, for KB QTL on chromosome 1BS, the LOD score value for the marginal analysis was 3.41 while for the individual analysis it was only 2.47. In the second QTL, on chromosome 2AS, the marginal LOD for Yr was 2.53 while the individual LOD was higher (2.75), and in both the cases these were significant. Also on chromosome 2DS at 9 cM position, both the marginal and the individual LOD score for KB were significant, however, in the individual analysis the LOD was higher (2.53 and 3.34, respectively).

In chromosome 5BL the behavior for KB was the opposite, the marginal LOD score (0.17) at 6 cM position was not significant, but the individual LOD score was significant (2.78). Similarly, on the chromosome 6DS again both marginal and individual LOD scores were non-significant but values for the marginal QTL was lower (0.03) than the individual one (2.26).

For TS, the marginal and individual LOD scores followed similar pattern as for KB. For example in chromosome 3AS, both marginal and individual LOD scores were significant with values 3.84 and 4.08, respectively. In summary, for KB and TS the marginal and individual analyses were very similar.

For Yr and Lr, in almost all the putative QTLs reported, the behavior of the marginal and individual analyses was the same, the marginal LOD scores were higher than the individual LOD scores, except for the QTL of chromosome 3AS for Yr (1.53 and 2.21, respectively). For the QTL detected in chromosome 5BL.1 for Lr with LOD scores values of 1.45 and 1.20 for marginal and individual traits, respectively. In the two QTLs in which Yr was found to be significant in the marginal analysis, in the chromosomes 2AS and 5BL, the marginal LOD scores were significant while the individual LOD scores were not (2.53 vs 1.30 and 3.00 vs 2.27, respectively). Also for Lr, the only QTL in which the marginal LOD score was found to be significant in the chromosome 6DS (5.53), the individual LOD was not significant (2.03).

### General behavior for individual QTL

The KB QTL on chromosome 1BS (*QKb.cimmyt-1BS*) was detected exactly at the position of the marker *Xgwm273*. The traits more influencing the joint profile were KB which had a profile very similar to the joint profile with a R^2^ = 0.11 (Fig. 2 A; Table 3). For traits KB, and Lr resistance alleles are contributed by the parent HD29 (negative sign), while for TS and Yr the trait enhancing alleles were contributed by parent WH542 (positive sign). Few rust resistance genes are reported in chromosome translocations 1BL.1RS and 1DL.1RS and introduced into wheat from ‘Imperial’ rye [39]. *Yr15* has been reported to be located on the short arm of chromosome 1B [45]. The *Lr46* was found tightly linked or pleiotropic to a stripe rust resistance gene designated *Yr29*. However marginal trait analysis could not detect QTLs associated with rusts resistance on chromosome 1BS.

It was observed that often while the additive effects for the individual traits were relatively high, for the joint analysis the additive effects were low (0.05). Due to the manner in which the identical by descent probabilities were calculated for the genetic predictors, a positive sign in the additive effect means that the allele which increments the numeric value of the traits comes from the parent WH542.

The QTL on chromosome 2AS (*QYr.cimmyt-2AS*) was found in between the markers *Xgwm122* and *Xppo33*, the nearest was *Xgwm122* (Table 3, Fig. 2B). The traits that most influence the joint profile were Yr followed by TS with R^2^ values of 0.04 and 0.24, respectively. Although Yr had a LOD score value greater than TS, the R^2^ values showed an opposite behavior, indicating the greater complexity of Yr disease as compared with TS. For KB, the R^2^ value was medium (0.18) while for Lr it was very low (0.01). Similar to previous cases, the additive sign for KB, Yr and Joint were negative, while for TS and Lr were positive. The additive value for the joint analysis was very small (0.01) related to the individual traits.

The most significant QTL detected on chromosome 2DS (*QKb.cimmyt-2DS*) was located near the marker *Xcfd53* (Fig. 2 C, Table 3). In the peak position for the joint profile (31 cM), the KB individual trait was significant. Also, around 9 cM position, the joint and marginal KB profiles had significant LOD values (4.27 and 2.53). The largest R^2^ values were for KB (0.16 and 0.13). Unlike the first two QTLs, the additive effect for TS and the joint analysis were both negative (parent WH542). Also for Yr the sign was opposite to the previous ones, while for KB were negative. The absolute value for the joint additive effect was now clearly greater than in the previous two QTLs.

The QTL found in chromosome 3AS (*QTs.cimmyt-3AS*) for individual trait TS was significant, and this trait influenced the joint profile the most. R^2^ value was 0.23 and positive additive as the joint effect (Fig. 2 D, Table 3). The markers flanking the peak were *Xbarc45* and *Xbem29* with *Xbarc45* being the nearest. The resistance QTL for TS identified on chromosome arm 3AS (*QTs.ksu-3AS*) has also been reported in a previously [29]. However, here we are reporting additional PCR-based marker (EST-STS) and the QTL flanked by markers *Xbem29* and *Xbarc45*, and will be useful for marker-assisted selection. Effertz et al. [42] reported the restriction fragment length polymorphism marker *Xcdo395* on chromosome 3AS with a portion of the insensitivity of Opata 85 to chlorosis-inducing crude culture filtrate of *P. tritici-repentis*. Our results confirm the association of the region (3AS) for a tan spot resistance. *QTs.ksu-3AS* could be the same because Opata 85 and WH542 share Jupateco and Bluejay in their pedigrees. This chromosomal region may be a source of MDR because it has also been reported to carry a QTL for resistance to Fusarium head blight in tetraploid wheat. Karnal bunt Yr, and Lr had negative (HD29) additive effect while for TS was positive (WH542).

The QTL *QYr.cimmyt-5BL.1* detected on the chromosome 5BL the more influencing trait was Yr which was the only individual significant trait with an R^2^ of 8.05% (Fig. 2 F, Table 3). It had a high positive additive effect for the joint profile as well. The peak for the joint profile was located in between the markers *Xgwm271* and *Xwmc235* with *Xgwm271* being the nearest marker. The highest R^2^ value was for TS (9.78%) and also with a positive (WH542) effect while KB had a negative (HD29) effect. Marker *Xfcp393* on the long arm of chromosome 5BL was significantly associated with resistance to TS and explained 27% of the phenotypic variation. A toxin insensitivity gene (*Tsn1*) in this interval has been reported previously in a mapping population from the cross of Chinese Spring (CS) and the CS-*T. dicoccoides* chromosome 5B disomic substitution line [46].

For the QTL found on the chromosome 6DS, the peak of the joint profile was located in between the markers *Xcfd42* and *Xcfd*49 with *Xcfd42* being the nearest (Fig. 2 G, Table 3). The more influencing as well as the only significant individual trait was Lr with an R^2^ value of 9.0% and a high positive additive effect. Now all the effects were positives except for KB and Yr however, Yr had a high negative effect.

In summary, KB and TS were the most significant traits in eight QTLs out of the thirteen. Yellow rust was found to be significant in three QTLs while Lr was significant in only one QTL. Total phenotypic variation explained by these thirteen QTLs for each disease resistance traits KB, TS, Yr, and Lr were 57%, 55%, 38%, and 23% respectively. The largest R^2^ values were found for KB and TS in almost all the QTLs. Usually the R^2^ values for Yr and Lr were very low except in the chromosome 3BL and 5BL for Yr and in chromosome 6DS for Lr. KB had negative (HD29 contributed all the allele to reduce the disease) additive effects in all the QTLs except for chromosome 3BL-1, while TS had positive (WH542 allele contributed to reduce the disease) effects for all QTLs except in chromosome 2DS-1, 2DS-2, and 3BL-1. Yr and Lr showed alternate positive and negative effects, Yr had negative effects in eight out the thirteen QTLs while Lr had positive effects in nine out the thirteen QTLs.

### Multi-environment QTL analysis

**Figure 3:**
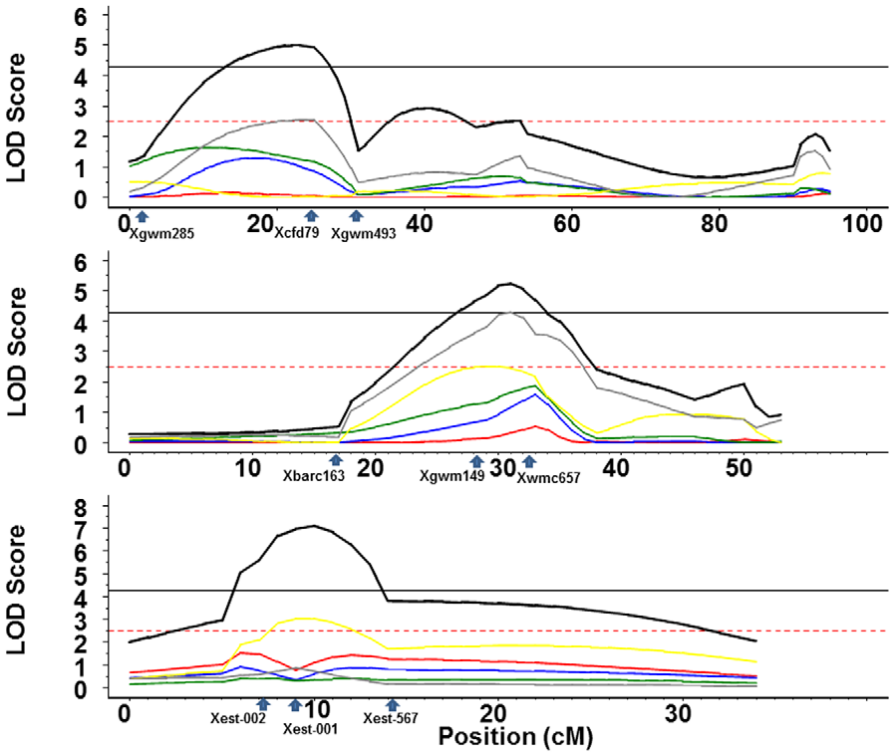
Multi-environment analysis and locations of QTL for resistance to Karnal bunt. (**A**) chromosome 3BS with markers *Xgwm285* (at 0 cM), *Xcfd49* (at 25 cM where the QTL is located), *Xgwm493* (at 31 cM), and *Xgwm108* (at 47 cM); (**B**) chromosome 4BL with marker *Xgwm495* (at 0 cM), *Xbarc163* (at 17 cM), *Xgwm149* (at 29 cM where the QTL is located), *Xwmc657* (at 33 cM), and *Xgwm513* (at 38 cM) and (**C**) chromosome 5DL with markers *X6augwm20* (at 0 cM), *Xest-ksm2* (at 5 cM), *Xest-002* (at 7 cM), *Xest-001*(at 9 cM where the QTL is located), and *Xest-567*(at 14 cM). LOD score is plotted against marker location. QTLs profiles legend with color red, blue, green, yellow, gray and black are KB01, KB02, KB03M, KB04M, KB05M, and joint effect respectively. Horizontal lines with black solid color mean threshold for Joint profile, and red dashed is threshold for marginal profiles.

The multi environment QTL analysis for KB, identified three putative QTLs, in chromosomes 3BS (*QKb.cimmyt-3BS*), 4BL (*QKb.cimmyt-4BL.1*), and 5DL (*QKb.cimmyt-5DL.1*) (Table 4). In general, the years KB05M and KB04M influenced joint analysis the most while KB01, KB02 and KB03M were not significant in any of the three QTLs. The individual and joint additive effects were all negatives except for KB04M in the chromosome 3BS and for KB05M in the chromosome 5DL. Two new QTLs, *QKb.cim-3BS.1* (Fig. 3 A) and *QKb.cim-5DL.1* (Fig. 3 C), with resistance alleles from HD29 were identified and mapped on chromosomes 3BS and 5DL. These explained 10 and 20% of the total phenotypic variation, respectively. A previously reported QTL, *Qkb.ksu-4BL.1*, was also identified on the same region of the chromosome and explained up to 13% of phenotypic variation. All the three QTLs were statistically significant in multiyear joint analyses. The QTL by environment was significant for the QTLs in the chromosomes 3BS and 5DL and non-significant in the chromosome 4BL.

**Table 4:** Significant QTLs and their positions in chromosomes for multi-environment analysis of Karnal bunt (KB) in five years (KB01M, KB02M, KB03M, KB04M, and KB05M).

LOD Score										
QTL	Chromosome	Position (cM)	Marker	KB01†	KB02	KB03M	KB04M	KB05M	JOINT‡	QxE¥
**1**	3BS	22	*Xcfd79*	0.07	1.10	1.30	0.00	2.53	5.00	3.44
**2**	4BL	31	*Xgwm149*	0.32	1.17	1.66	2.46	4.31	5.23	0.48
**3**	5DL	10	*Xest-001*	1.13	0.61	0.36	3.02	0.73	7.12	7.12

	Additive effects										R^2^
QTL	KB01	KB02	KB03M	KB04M	KB05M	JOINT	KB01	KB02	KB03M	KB04M	KB05M
**1**	−1.74	−4.10	−3.85	0.22	−3.46	−2.66	5.05	10.20	12.41	4.22	10.40
**2**	−3.33	−4.56	−5.24	−5.52	−4.94	−4.89	4.06	7.75	9.36	12.56	15.32
**3**	−4.52	−3.44	−2.32	−5.97	1.70	−0.08	11.06	9.52	8.94	20.27	0.35
R^2^ Total							14.27	19.17	21.72	27.28	24.10

† : Threshold for individual trait analysis is 2.5.

‡ : Threshold for Joint analysis is 4.27.

¥ : Threshold for Q×E Interaction for five environments is 2.40.

Values of the likelihood ratio test, the additive effect of the QTL, and the individual and total R^2^ values. For the individual trait profiles the LOD values are those found at the same position as the peak found for the joint profile.

### Specific behavior for individual QTL

The QTL (*QKb.cimmyt-3BS.1*), detected on the chromosome 3BS, had flanking markers *Xgwm285* and *Xcfd149* being *Xcfd149* the nearest (Fig. 3 A, Table 4). The trait with more influence in the joint profile was KB05M, the only one significant, with R^2^ value of 10.0% and negative additive effect (−3.46). In this QTL, all the additive effects were negative except for KB04M. Although KB02 and KB03M were not significant they had a high R^2^ value 10.0% and 12.0%, respectively, inclusive for KB03M the R^2^ value was greater than for KB05M (Table 4). This was because KB02, KB03M and KB05M showed similar trends and their maximum LOD score was reached near to the peak for the joint profile, while KB01 and KB04M showed a rather flat profile with very low LOD values along the entire chromosome (Fig. 3 A).

The QTL (*QKb.cimmyt-4BL.1*), on chromosome 4BL was associated to the markers *Xgwm149* and *Xwmc657*, *Xgwm149* being the nearest marker (Fig. 3 B, Table 4). Again, the more influencing individual trait was KB05M followed by KB04M which had a LOD value slightly lower than the threshold (Table 4). These two traits also had the highest R^2^ values 15.0% and 13.0%. In this QTL, all the individual and joint additive effects were negatives. Also in this QTL, all the individual traits showed similar trends in their profile along the entire chromosome and had their maximum LOD score very near to the joint peak. This QTL was the only in which the QTL x Environment test was not significant (Table 4).

For the QTL *QKb.cimmyt-5DL.1* found on chromosome 5DL, the markers flanking the joint peak were *Xest-wr001* and *Xest-567* being *Xest-wr001* the nearest (Fig. 3 C, Table 4). These flanking markers were used for cloning leaf rust resistance gene *Lr1*. *Lr1* is a dominant leaf rust resistance gene located on chromosome 5DL of bread wheat and the wild species *Aegilops tauschii* [47]–[48]. In present study, three polymorphic markers (*Xest-wr001*, *Xest-wr002*, and *Xest-wr003*) were used from resistance gene analogs (RGAs) clustering around the *Lr1* locus. These markers were used to map KB resistance QTLs on chromosome 5DL. This is most likely first KB resistance QTL mapped in wheat using a candidate gene approach and showed significant effect on the disease. More influencing trait and the only significant one was in year KB04M with a clearly high R^2^ value 20.0%. All the additive effects were negative except for KB05M. The behavior of all individual traits was similar as can be seen on their profiles reaching their maximum LOD scores values near to the joint peak.

It is not uncommon in wheat to find regions inherited as multi-disease resistance loci. These are typically due to absence of recombination from alien chromosomal segments, such as the stripe rust and mildew resistances from a rye chromosome 1RS segment or triple rust and nematode resistances from the *Ae. ventricosa* introgressed segment on wheat chromosome 2A [27], [49]. These introgressed segments were shown to carry diverse and multiple gene clusters that encode nucleotide binding and leucine rich repeat sequences, the most common class of plant disease resistance genes [49]–[50]. By contrast the *Lr34*/*Yr18*/*Pm38* locus of wheat has no history of alien introgression and thus suppressed recombination does not explain the multi-pathogen resistance found at this locus on wheat chromosome. Numerous wheat mapping studies, component parts or all of the multi-disease resistance traits on have been scored as a quantitative trait locus (QTL) partly due to the partial resistance phenotype and other rust resistance loci elsewhere in the wheat genome [15].

Delineating the locus to facilitate the molecular genetics characterization of the multi-disease resistance was boosted by the development of genetic stocks in the wheat genotypes Thatcher, Lalbahadur, Avocet and Arina from which ‘single gene’ families were generated. The partial resistance expression of the multi-pathogen resistance QTL was shown to be inherited as a simple Mendelian trait in the single gene families. In a few wheat backgrounds, such as Thatcher and its derivatives, the presence of *Lr34* enhances stem rust resistance. However, co-segregation of *Lr34* with the adult plant stripe rust resistance gene *Yr18* in exhibiting dual rust resistance in numerous wheat backgrounds may have contributed to the continued widespread use of the *Lr34*/*Yr18* germplasm in wheat breeding [15]. Subsequent observations that the *Lr34*/*Yr18* locus also contributed to partial resistance against adult plant powdery mildew (*Pm38*) highlighted the multi-pathogen nature of the *Lr34*/*Yr18*/*Pm38* locus in wheat chromosome [51] mutagenic changes to the ABC transporter alone were adequate to confer loss of the leaf rust, stripe rust and powdery mildew resistances encoded by *Lr34*/*Yr18*/*Pm38*. Together with haplotype analysis and high resolution mapping, it was established that a single gene, an ABC transporter, conferred all three resistances [15]. Strong parallels between the dual adult plant leaf and stripe rust resistance gene(s) *Lr46*/*Yr29* and *Lr34*/*Yr18* have been documented. Co-segregation of *Lr46*/*Yr29* with *Ltn2*, a second gene for leaf tip necrosis and adult plant powdery mildew partial resistance, *Pm39* [15] bear resemblance to the corresponding phenotypes of *Ltn1* and *Pm38* with the *Lr34*/*Yr18* gene. *Sr2* shows parallels with *Lr34* and *Lr46*, in that it is associated with multi-pathogen resistance. Tight linkage between *Sr2*, the leaf rust resistance gene *Lr27*, and partial APR to stripe rust (*Yr30*) and powdery mildew were observed [52]. Wheat plants with inactivated *Lr27* alleles from mutagenesis appear to have lost *Sr2* possibly indicating pleiotrophism [51]. In addition to the associated *Sr2* plant morphology with dark pigmentation or necrotic region on the peduncle and glumes often referred to as pseudo black chaff has remained inseparable from *Sr2* resistance in high resolution mapping [53].

From the results of the MT analysis described in this report, it can be observed that the joint effect of the combined analysis on the marginal effect for each trait reflects the correlation among the traits, which produce some increase or decrease in the individual effect. The combined MT QTL detection should be a more realist approach than individual QTL mapping analyses since physiological processes in plants always act as in a complex system and not in isolate individual effects. By taking into account the correlated structure of multiple traits, the joint analysis has several advantages, compared with separate individual analyses, for mapping QTL, including the expected improvement on the statistical power of the test for QTL and the precision of parameter estimation. In addition, the joint analysis provides formal procedures to test a number of biologically interesting hypotheses concerning the nature of genetic correlations between different traits. Further research is required to validate the QTLs with major effects as well as QTL regions with multi-pathogen resistance in different genetic backgrounds. Some of the resistances are affected mainly by additive effects. Thus a combination of major QTL in wheat breeding lines seems to be promising and more efforts to incorporate those QTLs that reveal resistance to different pathogens.

## References

[1] Brennan JP, Warham EJ, Hernandez J, Byerlee D, Cornel F (1990). Economic losses from KB of wheat in Mexico.. CIMMYT Economics Working Paper 90/02 CIMMYT, Mexico DF, Mexico.

[2] Rush CM, Stein JM, Bowden RL, Riemenschneider R, Boratynski T (2005). Status of Karnal bunt of wheat in the United States 1996–2004.. Plant Dis.

[3] Boshoff WHP, Pretorius ZA (1999). A new pathotype of *Puccinia striiformis* f. sp. *tritici* on wheat in South Africa.. Plant Dis.

[4] Singh RP, Hodson DP, Huerta-Espino J, Jin Y, Njau P (2008). Will stem rust destroy the world's wheat crop?. Adv Agron.

[5] Elias E, Cantrell RG, Hosford RM (1989). Heritability of resistance to tan spot in durum wheat and its association with other agronomic traits.. Crop Sci.

[6] Faris JD, Anderson JA, Francl LJ, Jordahl JG (1996). Chromosomal location of a gene conditioning insensitivity in wheat to a necrosis-inducing culture filtrate from *Pyrenophora tritici–repentis*.. Phytopathology.

[7] Faris JD, Anderson JA, Francl LJ, Jordahl JG (1997). RFLP mapping of resistance to chlorosis induction by *Pyrenophora tritici–repentis* in wheat.. Theor Appl Genet.

[8] Faris JD, Friesen TL (2005). Identification of quantitative trait loci for race non specific resistance to tan spot in wheat.. Theor Appl Genet.

[9] Singh S, Sharma I, Sehgal SK, Bains NS, Guo Z (2007). Molecular mapping of QTLs for Karnal bunt resistance in two recombinant inbred populations of bread wheat.. Theor Appl Genet.

[10] Bonin CM, Kolb FL (2009). Resistance to Fusarium head blight and kernel damage in a winter wheat recombinant inbred line population.. Crop Sci.

[11] Paillard S, Schnurbusch T, Tiwari R, Messmer M, Winzeler M (2004). QTL analysis of resistance to Fusarium head blight in Swiss winter wheat (*T. aestivum* L.).. Theor Appl Genet.

[12] Risser P, Ebmeyer E, Korzun V, Hartl L, Miedaner T (2011). Quantitative-trait loci for adult-plant resistance to *Mycosphaerella graminicola* in two large winter wheat populations.. Phytopathology.

[13] Dangl JJ, Jones JDG (2001). Plant pathogens and integrated defense responses to infection.. Nature.

[14] Poland JA, Balint-Kurti PJ, Wisser RJ, Pratt RC, Nelson RJ (2009). Shades of gray: the world of quantitative disease resistance.. Trends Plant Sci.

[15] Krattinger SG, Lagudah ES, Spielmeyer W, Singh RP, Huerta-Espino J (2009). A putative ABC transporter confers durable resistance to multiple fungal pathogens in wheat.. Science.

[16] Miedaner T, Risser P, Paillard S, Schnurbusch T, Keller B (2012). Broad-spectrum resistance loci for three quantitatively inherited diseases in two winter wheat populations.. Mol Breed.

[17] Faris JD, Li WL, Liu DJ, Chen PD, Gill BS (1999). Candidate gene analysis of quantitative disease resistance in wheat.. Theor Appl Genet.

[18] Cao H, Li X, Dong X (1998). Generation of broad-spectrum disease resistance by over expression of an essential regulatory gene in systemic acquired resistance.. Proc Natl Acad Sci USA.

[19] Wisser RJ, Kolkmanb JM, Patzoldta ME, Hollandc JB, Yu J (2011). Multivariate analysis of maize disease resistances suggests a pleiotropic genetic basis and implicates a GST gene.. PNAS.

[20] Zwonitzer JC, Zwonitzer JC, Coles ND, Krakowsky MD, Arellano C (2010). Mapping resistance quantitative trait Loci for three foliar diseases in a maize recombinant inbred line population—Evidence for multiple disease resistance?. Phytopathology.

[21] Bozkurt TO, McGrann GRD, MacCormack R, Boyd LA, Akkaya MS (2010). Cellular and transcriptional responses of wheat during compatible and incompatible race–specific interactions with *Puccinia striiformis* f. sp. *tritici*.. Mol Plant Pathol.

[22] Breseghello F, Sorrells ME (2006). Association mapping of kernal size and milling quality in wheat (*Triticum aestivum* L.) cultivars.. Genetics.

[23] Lagudah ES, Krattinger SG, Herrera-Foessel S, Singh RP, Huerta-Espino J (2009). Gene-specific markers for the wheat gene *Lr34/Yr18/Pm38* which confers resistance to multiple fungal pathogens.. Theor Appl Genet.

[24] Lin F, Chen XM (2009). Quantitative trait loci for non-race specific, high-temperature adult-plant resistance to stripe rust in wheat cultivar express.. Theor Appl Genet.

[25] Luo P, Hu X, Zhang H, Ren Z (2009). Genes for resistance to stripe rust on chromosome 2B and their application in wheat breeding.. Prog Nat Sci.

[26] William HM, Trethowan R, Crosby–Galvan EM (2007). Wheat breeding assisted by markers: CIMMYT's experience.. Euphytica.

[27] McIntosh RA, Wellings CR, Park RF (1995). Wheat rusts: an atlas of resistance genes.. CSIRO, Melbourne, Australia.

[28] Peterson RF, Campbell AB, Hannah AE (1948). A diagrammatic scale for estimating rust intensity of leaves and stems of cereals.. Can J Res.

[29] Singh S, Bockus WW, Sharma I, Bowden RL (2008). A novel source of resistance in Wheat to *Pyrenophora tritici repentis* race 1.. Plant Dis.

[30] Lander ES, Green P, Abrahamson J, Barlow A, Daly MJ (1987). MAPMAKER: an interactive computer package for constructing primary genetic linkage maps of experimental and natural populations.. Genomics.

[31] GenStat® Release 13 (2010). VSN International, Hemel Hempstead..

[32] Genstat 5 Committee (2005). Genstat for Windows Release 12.0.. VSN International, Wilkinson House.

[33] Malosetti M, Ribaut JM, Vargas M, Crossa J, van Eeuwijk FA (2008). A multi-trait multi environment QTL mixed model with an application to drought and nitrogen stress trials in maize (*Zea mays* L.).. Euphytica.

[34] Li J, Ji L (2005). Adjusting multiple testing in multilocus analyses using the eigenvalues of a correlation matrix.. Heredity.

[35] Lander ES, Botstein D (1989). Mapping Mendelian factors underlying quantitative traits using RFLP maps.. Genetics.

[36] Jiang CJ, Zeng ZB (1997). Mapping quantitative trait loci with dominant and missing markers in various crosses from two inbred lines.. Genetica.

[37] Basten CJ, Weir BS, Zeng ZB (2004). QTL Cartographer, Version 1.17.. Department of Statistics, North Carolina State University, Raleigh, NC.

[38] Churchill GA, Doerge RW (1994). Empirical threshold values for quantitative trait mapping.. Genetics.

[39] Malosetti M, Voltas J, Romagosa I, Ullrich SE, van Eeuwijk FA (2004). Mixed models including environmental covariables for studying QTL by environment interaction.. Euphytica.

[40] Piepho HP (2000). A mixed–model approach to mapping quantitative trait loci in barley on the basis of multiple environment data.. Genetics.

[41] Malosetti M, Boer MP, Bink MCAM, van Eeuwijk FA (2006). Multi-trait QTL analysis based on mixed models with parsimonious covariance matrices. In, Proceedings of the 8th World Congress on Genetics Applied to Livestock Production, August 13–18, Belo Horizonte, MG, Brasil.. http://www.wcgalp8.org.br/wcgalp8/.

[42] Effertz RJ, Meinhardt SW, Anderson JA, Jordahl JG, Francl LJ (2002). Identification of a chlorosis-inducing toxin from *Pyrenophora tritici-repentis* and the chromosomal location of an insensitivity locus in wheat.. Phytopathology.

[43] Miya A, Albert P, Shinya T, Desaki Y, Ichimura K (2007). CERK1: a LysM receptor kinase, is essential for chitin elicitor signaling in Arabidopsis.. Proc Natl Acad Sci.

[44] Wan J, Zhang X-C, Neece D, Ramonell KM, Clough S (2008). A LysM receptor-like kinase plays a critical role in chitin signaling and fungal resistance in Arabidopsis.. Plant Cell.

[45] Brown JKM, Hovmøller MS (2002). Aerial dispersal of pathogens on the global and continental scales and its impact on plant disease.. Science.

[46] Haen KM, Lu HJ, Friesen TL, Faris JD (2004). Genomic targeting and high-resolution mapping of the *Tsn1* gene in wheat.. Crop Sci.

[47] Cloutier S, McCallum B, Loutre C, Banks TW, Wicker T (2007). Leaf rust resistance gene *Lr*, isolated from bread wheat (*Triticum aestivum* L.) is a member of the large psr567 gene family.. Plant Mol Biol.

[48] Jansen RC, Stam P (1994). High resolution of quantitative traits into multiple loci via interval mapping.. Genetics.

[49] Seah S, Spielmeyer W, Jahier J, Sivasithamparam K, Lagudah ES (2000). Families of resistance gene sequences within an introgressed chromosomal segment derived from *T. ventricosum* in wheat which confer resistance to nematode and rust pathogens.. Mol Plant-Microbe Interact.

[50] Mago R, Spielmeyer W, Lawrence GJ, Ellis JG, Prior AJ (2004). Resistance genes for rye stem rust (*SrR*) and barley powdery mildew (*Mlo*) are located in syntenic regions on short arm of chromosome.. Genome.

[51] Spielmeyer W, McIntosh RA, Kolmer J, Lagudah ES (2005). Powdery mildew resistance and *Lr34/Yr18* genes for durable resistance to leaf and stripe rust cosegregate at a locus on the short arm of chromosome 7D of wheat.. Theor Appl Genet.

[52] Singh RP, Mcintosh RA (1984). Complementary genes for reaction to *Puccinia recondite tritici* in *Triticum aestivum*. 1. Genetic and linkage studies.. Can J Genet Cytol.

[53] Kota R, Spielmeyer W, McIntosh RA, Lagudah ES (2006). Fine genetic mapping fails to dissociate durable stem rust resistance gene *Sr2* from pseudo black chaff in common wheat (*Triticum aestivum* L).. Theor Appl Genet.

